# PomBase 2015: updates to the fission yeast database

**DOI:** 10.1093/nar/gku1040

**Published:** 2014-10-31

**Authors:** Mark D. McDowall, Midori A. Harris, Antonia Lock, Kim Rutherford, Daniel M. Staines, Jürg Bähler, Paul J. Kersey, Stephen G. Oliver, Valerie Wood

**Affiliations:** 1 European Molecular Biology Laboratory, European Bioinformatics Institute (EMBL-EBI), Wellcome Trust Genome Campus, Hinxton, Cambridgeshire CB10 1SD, UK; 2 Cambridge Systems Biology and Department of Biochemistry, University of Cambridge, Sanger Building, 80 Tennis Court Road, Cambridge, Cambridgeshire CB2 1GA, UK; 3 Research Department of Genetics, Evolution and Environment, and UCL Cancer Institute, University College London, Darwin Building, Gower Street, London WC1E 6BT, UK

## Abstract

PomBase (http://www.pombase.org) is the model organism database for the fission yeast *Schizosaccharomyces pombe*. PomBase provides a central hub for the fission yeast community, supporting both exploratory and hypothesis-driven research. It provides users easy access to data ranging from the sequence level, to molecular and phenotypic annotations, through to the display of genome-wide high-throughput studies. Recent improvements to the site extend annotation specificity, improve usability and allow for monthly data updates. Both in-house curators and community researchers provide manually curated data to PomBase. The genome browser provides access to published high-throughput data sets and the genomes of three additional *Schizosaccharomyces* species (*Schizosaccharomyces cryophilus*, *Schizosaccharomyces japonicus* and *Schizosaccharomyces octosporus*).

## INTRODUCTION

The fission yeast *Schizosaccharomyces pombe* is a unicellular eukaryote that has been used as a model organism for studying a diverse array of biological processes, from the cell cycle to signaling, for over 60 years (1). It was the sixth eukaryotic organism to have its genome completely sequenced (2). With a thriving community generating data from small and large-scale projects, a central hub to curate and integrate information is vital to facilitate data interpretation and hypothesis generation, and to guide further research.

PomBase (http://www.pombase.org) was launched in 2011 as the model organism database for fission yeast (3). The PomBase portal provides centralized access to gene- and genome-scale information, emphasizing data acquired by manual literature curation. In a novel community curation initiative, fission yeast researchers now contribute significantly to gene annotation, using the Canto online curation tool (4).

PomBase presents information in gene-specific pages that include summary data on each gene and its product, such as its biological functions, cellular localization, phenotype data, modifications, interactions, regulation and gene expression.

PomBase offers a customized Ensembl Genome browser (5) to provide access to the genome sequence and features, and to visualize high-throughput data sets in a genomic context.

## BIOLOGICAL DATA

PomBase curators focus on extracting data from historical papers and on providing help and guidance to researchers who curate their own papers using Canto. The inclusion of genome-scale datasets has resulted in a large increase in the volume of data curated.

### High-throughput datasets

**Figure 1.**
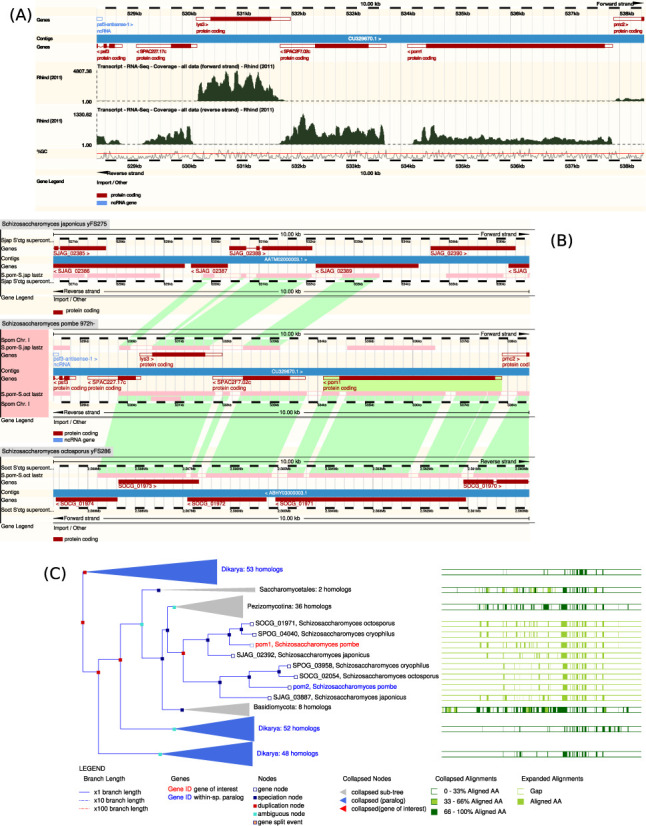
Views available in the genome browser. (**A**) Region display with two tracks enabled, displaying RNA-Seq coverage data (12). The two tracks show the forward (top) and reverse (bottom) strand reads along with the genes that are mapped to the region. (B) Region comparison display showing the regions of alignment between *Schizosaccharomyces pombe* and *Schizosaccharomyces japonicus* (top) and *Schizosaccharomyces octosporus* (bottom) in green. (C) Gene tree view generated using the Compara framework, with the gene of interest, *pom1* (SPAC2F7.03c), highlighted in red.

PomBase gene pages typically include data from various types of large-scale experiments, such as gene expression data (6,7), phenotypic analysis (8,9) and interaction data (10). Within the genome browser, PomBase hosts sequence-based datasets from a variety of high-throughput experimental techniques, such as nucleosome positioning (11), transcriptomic data (see Figure 1A) (6,11–12), replication profiling (13), polyadenylation sites (14,15) and chromatin binding (16). The datasets included to date are those requested by the fission yeast community, and for which the publication authors have provided data to PomBase.

### Additional species

In addition to *S. pombe*, the genomes of *Schizosaccharomyces cryophilus*, *Schizosaccharomyces japonicus* and *Schizosaccharomyces octosporus* (12) are now accessible via the genome browser, as the result of collaboration with the Ensembl Genomes project (17). All-against-all DNA alignments between the four *Schizosaccharomyces* species can also be displayed in the PomBase browser (see Figure 1B). The new *Schizosaccharomyces* species are among the 52 fungal species included in a protein multiple sequence alignment (see Figure 1C). *S. pombe* also represents the fission yeast clade in protein multiple sequence alignments of a further set of 178 species covering a broad taxonomic range including human and bacteria, which are visible in the genome browser.

## INFRASTRUCTURE AND PROCEDURAL IMPROVEMENTS

Manually curated data is stored within a Chado relational database (18). During the release procedure, a snapshot of the curation database is created and the annotations are imported into an Ensembl schema on a MySQL database. The Ensembl schema provides the back-end architecture for the genome browser and houses the annotations for the PomBase site.

The import pipeline that transfers data from the PomBase Chado curation database to the Ensembl database has been altered to accommodate increasing annotation complexity (as described below). Update procedures have been improved to implement data consistency checks on the database and to use the Selenium testing framework (http://www.seleniumhq.org) on the web interface. This more robust infrastructure has enabled PomBase to implement a monthly release cycle. Improved back-end data storage and retrieval has reduced the gene page loading time, even as the amount of data presented has increased, enabling users to navigate through multiple genes with minimal delays.

## INTEGRATING AND VISUALIZING DATA

To maintain a readable display of increasingly complex data, and incorporate new data types, there have been major improvements to the organization and presentation of data on the gene pages. The most extensive changes have affected three key regions of the gene page: the displays of Gene Ontology (GO) (19,20) annotations, Fission Yeast Phenotype Ontology (FYPO) annotations (21) and gene expression data. More subtle changes have also been introduced throughout the gene pages.

### Ontology annotations and extensions

The most significant change affecting annotation complexity in PomBase is the introduction of ‘annotation extensions’ that increase the expressivity of annotations to ontology terms. With active participation by PomBase curators, the GO Consortium introduced annotation extensions in 2013 (22) to enable curators to capture additional contextual details such as effector–target relationships and temporal or spatial aspects of biological processes. Whereas previously each GO annotation combined a single gene product with a single GO term, and was independent from any other GO annotations, extended annotations can capture interconnections between multiple annotations as well as links to additional ontologies.

Each annotation extension consists of a relation and an identifier that refers to another ontology term (GO, SO (23), ChEBI (24), PSI-MOD (25), etc.) or another gene. An annotation may have one or more extensions, each with its own relationships and sources, and ‘compound’ extensions can be made by combining single extensions.

To date, PomBase curators have added extensions to over 2000 GO annotations. PomBase has also adopted the annotation extension model for phenotype (FYPO) and gene expression annotations, as described below. Table 1 shows the total number of annotations in PomBase as of August 2014, and the number that have extensions, for four ontologies plus gene expression.

**Table 1.** Summary of annotations and extensions in PomBase as of September 2014

Curated data type	Annotation	Annotation	Gene
	count	extensions	coverage
Gene expression	40 403	40 403	7017
Phenotype (FYPO)	36 382	11 722	4942
Gene Ontolgy (GO)	37 224	2149	5301
Modifications (MOD)	11 265	7255	2009
Protein sequence	943	N/A	764
Features (SO)			

Annotation Count: total number of annotations of each type, including those with extensions; Annotation extensions: number of annotations that have one or more extensions apiece; Gene coverage: number of genes that have at least one annotation of the given type.

**Figure 2.**
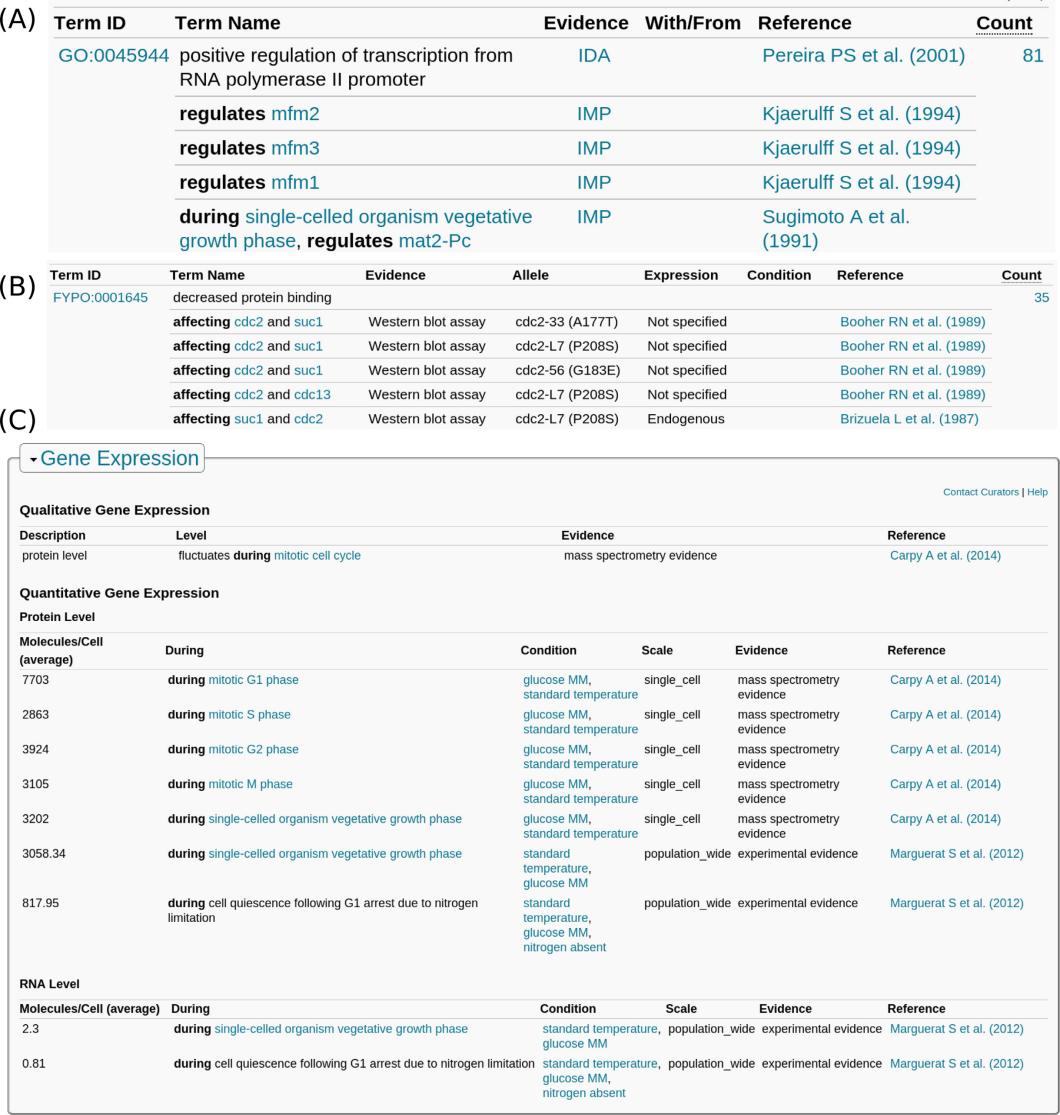
PomBase gene page views for example annotations: (**A**) GO (gene *ste11* - SPBC32C12.02); (**B**) FYPO (gene *cdc2* - SPBC11B10.09); (**C**) Gene expression (gene *clr3* - SPBC800.03). These examples highlight the display of annotation extensions and their use within the context of different ontologies.

To accommodate annotation extensions, PomBase has adapted its Chado and Ensembl relational database schemata and loading procedures and enhanced the gene page ontology annotation displays. On PomBase gene pages, annotation extensions are shown in rows below the ontology term, with the relevant evidence code and annotation source. Identifiers and relation strings are converted to human-friendly text, such as a gene name or ontology term, wherever possible. For example, Figure 2A shows annotations to GO:0045944 from the *ste11* (SPBC32C12.02) gene page. Annotations without extensions are displayed first, followed by those with extensions, and the bottom row shows a compound annotation extension (26).

Phenotype annotations have also considerably increased both in number and complexity. FYPO annotations include allele details, expression level, experimental conditions, the evidence and source, and annotation extensions that represent penetrance and severity. Phenotype annotation extensions also capture specific genes used in assays for phenotypes such as protein localization or gene expression level. On each gene page, FYPO annotations are grouped by whether the phenotype is relevant at the level of a cell population or an individual cell, and display annotation extensions similarly to the GO tables. Figure 2B shows alleles of *cdc2* (SPBC11B10.09) that have been annotated to ‘decreased protein binding’ (FYPO:0001645), affecting several other gene products.

### Targets

A new table on the gene pages, ‘Target Of’, reports effects of other genes on the gene of interest, such as modification or regulation. ‘Target Of’ annotations are the reciprocal of GO and FYPO annotation extensions. The ‘Target Of’ display includes the relevant gene, a relationship and the annotation source. For example, *cdc2* (SPBC11B10.09) is annotated as the substrate of *csk1* (SPAC1D4.06c) protein kinase activity (27) and *cdc25* (SPAC24H6.05) protein phosphatase activity (28).

### Gene expression

Quantitative gene expression data have been imported from two large datasets covering the expression of 3175 (7) and 7016 (6) gene products. Gene expression annotations may include extensions indicating that the expression level was measured in a specific phase of the cell cycle or under specific growth conditions. Further qualitative data have also been manually imported into PomBase from the literature. When available, this information is displayed on the gene pages, providing details of the experimental conditions, evidence, scale and source. Figure 2C shows the display of gene expression data for *clr3* (SPBC800.03).

### Data visualization in genome browser

In the PomBase genome browser, data tracks are presented with curated metadata, links to the relevant publication via the Europe PMC portal (29) and, where appropriate, links to external source databases. Users have the option of viewing their own data privately within the context of the genome browser, or submitting data to be hosted by PomBase for public viewing.

## OTHER IMPROVEMENTS

PomBase now offers a motif finder that can retrieve lists of genes that match a particular protein sequence pattern. In the PomBase advanced search, the interfaces for constructing custom queries and retrieving results have been enhanced.

User-experience testing conducted after the initial PomBase release identified several opportunities for usability improvements. Accordingly, changes to the navigation and organization of the gene pages, such as collapsible intra-page menus, now make data more intuitively visible. Interfaces requiring user interaction are now also more intuitive.

## OUTREACH AND USAGE

PomBase includes documentation for all gene page sections, links to Ensembl documentation for the genome browser and a Frequently Asked Questions section. Various web forms offer convenient links for users to contact curators to ask questions or submit high-throughput datasets to be included in the genome browser or on the gene pages. PomBase curators invite all authors of new fission yeast publications to curate their own papers using Canto. PomBase also sends announcements and help to a dedicated mailing list and to various social media outlets including Twitter (@PomBase), LinkedIn (http://www.linkedin.com/company/pombase) and Google+ (+PombaseOrg).

## FUTURE DIRECTIONS

Canto and the gene pages will be extended to support the curation and display, respectively, of multiple-gene phenotypes (double mutants, triple mutants, etc.). Work has also begun to create pages for non-gene sequence features, such as the centromeres, which at present can only be viewed in the genome browser.
